# Multicomponent Polyphenolic Extracts from *Vaccinium corymbosum* at Lab and Pilot Scale. Characterization and Effectivity against Nosocomial Pathogens

**DOI:** 10.3390/plants10122801

**Published:** 2021-12-17

**Authors:** Eva Gato, Astrid Perez, Alicja Rosalowska, Maria Celeiro, German Bou, Marta Lores

**Affiliations:** 1 Departamento de Microbiología, Complejo Hospitalario Universitario A Coruña (CHUAC), Instituto de Investigación Biomédica A Coruña (INIBIC), Universidad de A Coruña (UDC), CIBER de Enfermedades Infecciosas (CIBERINFEC), Instituto de Salud Carlos III, 15006 A Coruña, Spain; eva.gato.corral@sergas.es (E.G.); german.bou.arevalo@sergas.es (G.B.); 2 LIDSA, Department of Analytical Chemistry, Nutrition and Food Science, Universidade de Santiago de Compostela, 15782 Santiago de Compostela, Spain; ally.rosalowska@gmail.com; 3 CRETUS, Department of Analytical Chemistry, Nutrition and Food Science, Universidade de Santiago de Compostela, 15782 Santiago de Compostela, Spain; maria.celeiro.montero@usc.es

**Keywords:** blueberries, polyphenols, bactericidal effects, antimicrobial resistance, green extraction techniques, liquid chromatography–tandem mass spectrometry, medium-scale ambient temperature systems

## Abstract

An extraction method was designed and scaled up to produce multicomponent polyphenolic extracts from blueberries (*Vaccinium corymbosum*) of three different varieties. The process was specifically drawn up to comply with green chemistry principles. Extracts were obtained for the direct assessment of their antimicrobial and antiadhesive activities, and their direct use in the control of infections caused by concerning multidrug-resistant nosocomial pathogens. Analytical characterization was performed by liquid chromatography–tandem mass spectrometry (LC–MS/MS). Similar qualitative profiles were obtained in the three studied varieties with some significant quantitative differences. Up to 22 different polyphenols were identified with a clear predominance of anthocyani(di)ns followed by flavanols, non-flavonoids, and far behind by flavan-3-ols and procyanidins. The individual content of the main polyphenols was also discussed. A pilot scale extract has been also produced as a proof-of-concept, showing that scaling-up triples the content of bioactive phytochemicals. The effect of the polyphenolic extracts was analyzed against seven multidrug-resistance bacterial species by performing biofilm formation and growth and killing curves assays. All the studied varieties showed antibacterial and antiadhesive activities, being the extract containing the highest concentration of bioactive polyphenols, the most active with a high bactericidal effect.

## 1. Introduction

Nowadays, the rapid emergence of multidrug-resistant pathogens represents one of the major medical challenges. The gradual loss of effective classical antibiotics for many bacterial pathogens, combined with the slowing development of new antibiotics, elevates the need for novel innovative therapies. Effective drugs for infections caused by tough-to-treat pathogens, that are less prone to causing resistance are required. Until recently, carbapenems have been considered as last-line antibiotics for the treatment of infections caused by multidrug-resistant (MDR) Gram-negative pathogens. However, several carbapenem resistant (CR) pathogens are increasing, representing a great threat to human health [1,2,3].

The antimicrobial resistance of these pathogens can even increase when they grow in biofilms, which are organized communities of bacteria encased in a self-produced matrix made of extracellular polymeric substances [4]. Biofilm-associated bacteria protect themselves from host defence, disinfectants, and antibiotics. Biofilms can be found in both abiotic and biotic surfaces, such as different tissues and medical devices. The contamination of medical devices can result in biofilm formation, increasing the risk of nosocomial infections. Moreover, due to the high resistance to antimicrobials shown by biofilm-associated bacteria, these device-related infections are difficult to treat, being responsible for recurrence of infections [4,5].

Antimicrobial activities have been mainly studied for cranberry proanthocyanidins, the basis of widely used medicines that exploit their anti-adherent capacity and inhibit uropathogenic bacteria [6,7,8,9,10,11]. On the other hand, blueberry polyphenols have been less studied [12,13,14]. Their extracts have been obtained in organic solvents and further purified to be assessed against a number of bacteria [12,13] or combined with other berry extracts (e.g., blackberries) for studies related to foodborne microorganisms [14].

The search for biologically active compounds of plant origin against bacterial biofilms, ubiquitous in natural, clinical and industrial environments, is a current trend [15,16]. Natural compounds that target microbial virulence factors, such as biofilm formation, or resistance determinants, can be successful strategies for controlling infections caused by multidrug-resistant (MDR) pathogens. To this end, chemical therapies based on natural products from plants phytoarsenals have emerged as an excellent option [16,17,18]. Polyphenols stand out among this phytochemical arsenal of secondary metabolites. The antibiofilm activity of some polyphenolic extracts of plant origin have been reported [16,18,19,20]. Anthocyanins are the most abundant polyphenolic group of compounds in blueberry extracts, followed by flavonols, phenolic acids, flavan-3-ols, and procyanidins; although this order can vary depending on the variety, the geographical origin, the characteristics of the soil in which it is grown, the degree of maturity, or even whether it is wild, conventionally grown, or organically grown.

Based on the knowledge outlined above, extracts obtained from three different *Vaccinium corymbosum* varieties, VcV (Ventura), VcS (Star), and VcE (Emerald), were tested to assess the activity against one of the major bacterial virulence factors (biofilm formation) in the most concerning nosocomial pathogens in terms of microbial resistance. The survival potential of the target microorganisms treated with the obtained blueberry natural extracts was also evaluated. The genus *Vaccinium* is rich in different families of polyphenols, in which antimicrobial activities have already been demonstrated [18]. Plants synthesize this wide range of secondary metabolites as a defensive chemical arsenal against the attack of very diverse microorganisms, a defence that cannot be faced with a single chemical family.

The extracts obtention procedures should follow the green analytical chemistry (GAC) principles [21] and be easily scalable, to become a realistic alternative to be applied beyond the laboratory scale. The desired multicomponent extract must be obtained in a solvent compatible with the antimicrobial activity tests (antibiofilm and growth and killing curves) and be safe to use on surfaces and medical devices coming into contact with humans, even in an invasive manner (for instance, catheters and respirators). The most employed sample preparation methods to obtain polyphenols from raw materials of plant origin have been solid–liquid extraction [22,23,24], assisted by ultrasound energy (UAE) [25,26,27,28,29] in some cases. Recently, other procedures based on electrically assisted methods, such as high voltage electrical discharges (HVED) and pulsed electric field technology (PEF) [30], have been reported. The solvents used to extract berry polyphenols traditionally ranged from hydro-organic mixtures [25,26,27,29] to pure solvents [22,23,24,28].

In this work, the chosen system was a medium scale ambient temperature (MSAT) setup, originally designed to obtain polyphenols from white grape marc as an intermediate step to the final pilot scale and patent-protected process [31]. The MSAT system has been successfully applied to extract polyphenols from Scotch broom (*Cytisus scoparius*) [32]. This configuration easily allows the further scale-up to pre-industrial production. In addition, it meets the green analytical chemistry (GAC) principles, specifically with the sixth principle (Design for Energy Efficiency [21]), since it is conducted at an ambient temperature and pressure with a minimum energy consumption. The use of water enriched with mineral salts, which improves the extraction performance for plant phenolics and complies with the fifth principle of green chemistry (Safer Solvents and Auxiliaries [21]), has been proposed.

Therefore, the obtained extract also meets the GAC requirements, its antibacterial activities can be directly assessed, and, at the same time, it can be directly used in products for the disinfection of surfaces and materials intended for use in human medicine. Moreover, it can be easily freeze dried if necessary. The MSAT extraction procedure has been applied not only to the premium commercial fruit, but also to surplus fruit from the production companies. The idea was to be able to process non-grading, rejected, or bulk fruits, which remains after the best, largest, or flawless ones have been chosen, i.e., they are “ugly fruit” from a commercial point of view, but ideal for the application proposed in this work. Residues from the agri-food industry that processes blueberries for juice, yogurt, and other derivatives can also be used as source material. This aspect complies with the seventh principle of green chemistry: Use of Renewable Feedstocks, which implicitly contemplates the reuse of rejects and by-products, in line also with the trend towards a circular economy that is and must be a growing trend in the agri-food sector [21].

Therefore, the main goal of this work is the assessment of the antibacterial activities of the green extracts obtained from different *Vaccinium corymbosum* varieties, VcV, VcS, and VcE, employing a MSAT procedure. An extract at a pre-industrial scale, applying the patented extraction process for other agri-food processing by-products [31], was also obtained. All of them were analytically characterized by liquid chromatography–tandem mass spectrometry (LC–MS/MS) to elucidate their polyphenolic profile, and their quantitative composition was compared.

## 2. Results

### 2.1. Analytical Characterization of MSATs Blueberry Extracts

In a first approach, the total polyphenol content (TPC) of the extract was evaluated (see experimental procedure in Section 4.4.), which highlighted the content of the Ventura (VcV) blueberries (343 ± 10 mg GAE L^−1^) compared to the Emerald (VcE) (128 ± 5 mg GAE L^−1^) and Star (VcS) (163 ± 12 mg GAE L^−1^) ones.

The individual polyphenols were analyzed by LC–MS/MS for the three extracts. Results are shown in Table 1. Compounds belonging to three families of non-flavonoid polyphenols and four families of flavonoids were detected, with the latter accounting for 92% to 97% in the three varieties studied. The low proportion of non-flavonoid polyphenols can, however, lead to misinterpreting the abundance of individual polyphenols, as chlorogenic acid is one of the most abundant compounds, particularly in the Ventura and Star varieties.

Additionally, the pulp and peel of the variety that presented the highest polyphenols concentration, VcV, were also extracted by MSATs and the TPC was evaluated, to increase the knowhow on feedstock for the eventual availability of such blueberry processing wastes. As can be expected, the peel of the blueberry (454 ± 75 mg GAE L^−1^) presented higher TPC than the pulp (124 ± 16 mg GAE L^−1^), considering that more than 95% of the total anthocyanins are found precisely in the peel; however, the pulp still preserves a significant amount of polyphenols.

### 2.2. Antibacterial Activities of Blueberry Extracts

#### 2.2.1. Antimicrobial Effect

**Figure 1:**
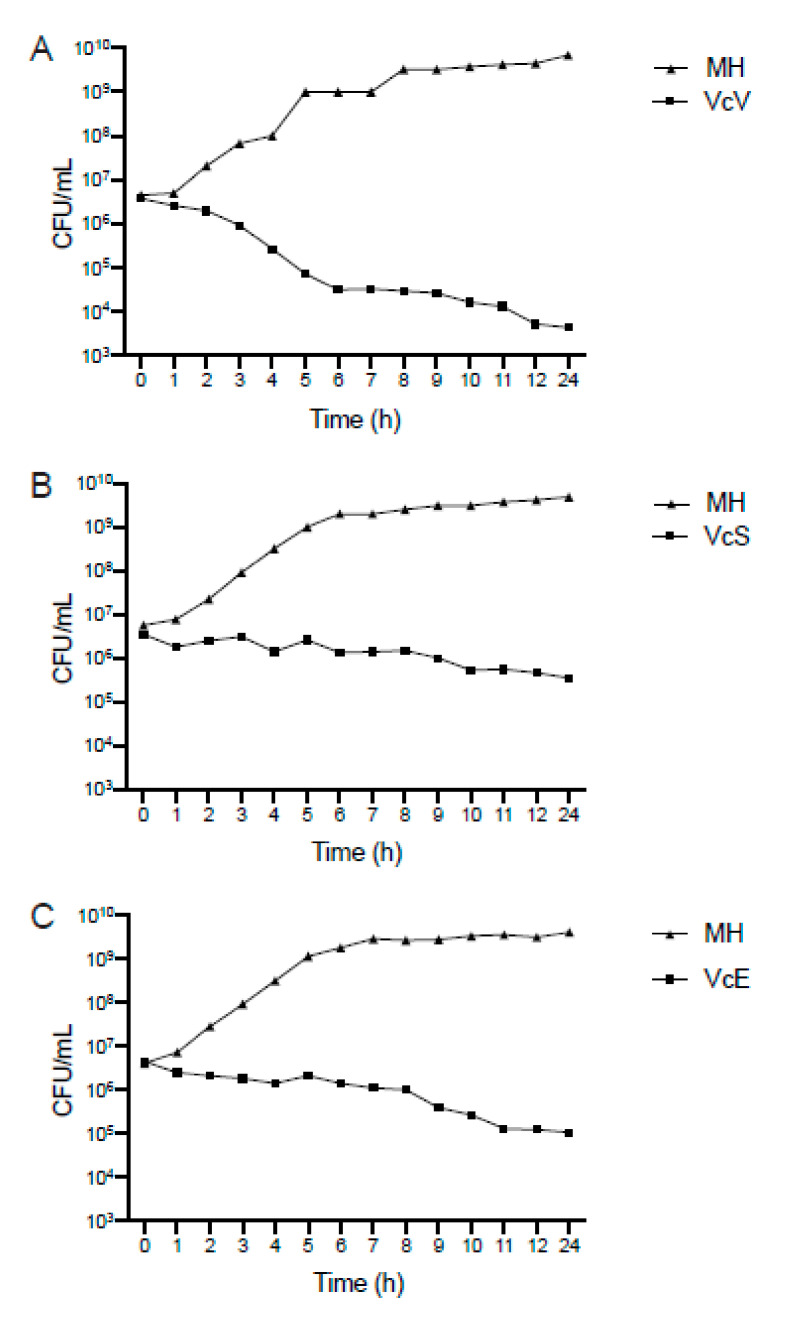
Time-killing curve analysis: (**A**) VcV (104.9 μg mL^−1^ of bioactive polyphenols); (**B**) VcS (40.6 μg mL^−1^ of bioactive polyphenols); and (**C**) VcE (40.3 μg mL^−1^ of bioactive polyphenols). MH: control group (absence of extract). The data corresponds to the median of three biological replicates.

The inhibitory and bactericidal activity of the three saline blueberry extracts, VcV, VcS, and VcE, were analyzed on seven bacterial strains that are considered the most concerning pathogenic species in terms of antibiotic resistance by the World Health Organization (WHO) [2]. In a first step, the bactericidal effect of the three phenolic extracts was tested against an epidemic clone of carbapenem-resistant *K. pneumoniae*. The results are depicted in Figure 1.

**Figure 2:**
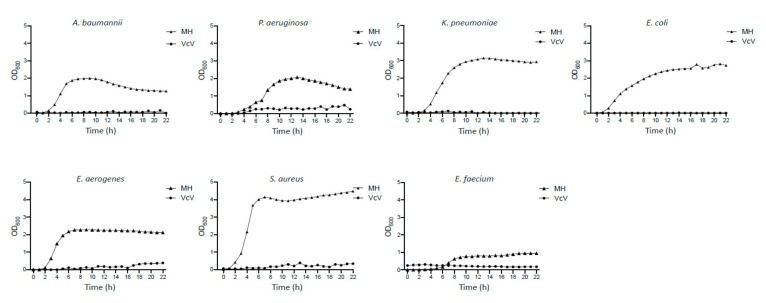
Growth curves of the bacterial strains indicated in the presence of the blueberry extract VcV (104.9 μg mL^−1^). MH: control group (absence of extract). The data corresponds to the mean of the three biological replicates.

As it is shown in Figure 1, the time-killing curve analysis revealed that only the VcV extract induced a bactericidal effect in *K. pneumoniae* as the bacterial population decreased 3 log10, when incubated in the presence of the VcV (bioactive polyphenols: 104.9 μg mL^−1^) extract over 24 h (Figure 1A). However, when the pathogen *K. pneumoniae* was treated with VcS (bioactive polyphenols: 40.6 μg mL^−1^) or VcE (bioactive polyphenols: 40.3 μg mL^−1^) extracts, the effect was bacteriostatic, as the reduction in the bacterial population was not enough to be considered a bactericidal activity (Figure 1B,C). Since the best activity was achieved by the VcV extract, its efficacy against six other concerning multidrug-resistant pathogens were analyzed. Figure 2 summarizes the obtained results from the bacterial growth inhibitory analysis.

#### 2.2.2. Antibiofilm Effect

**Figure 3:**
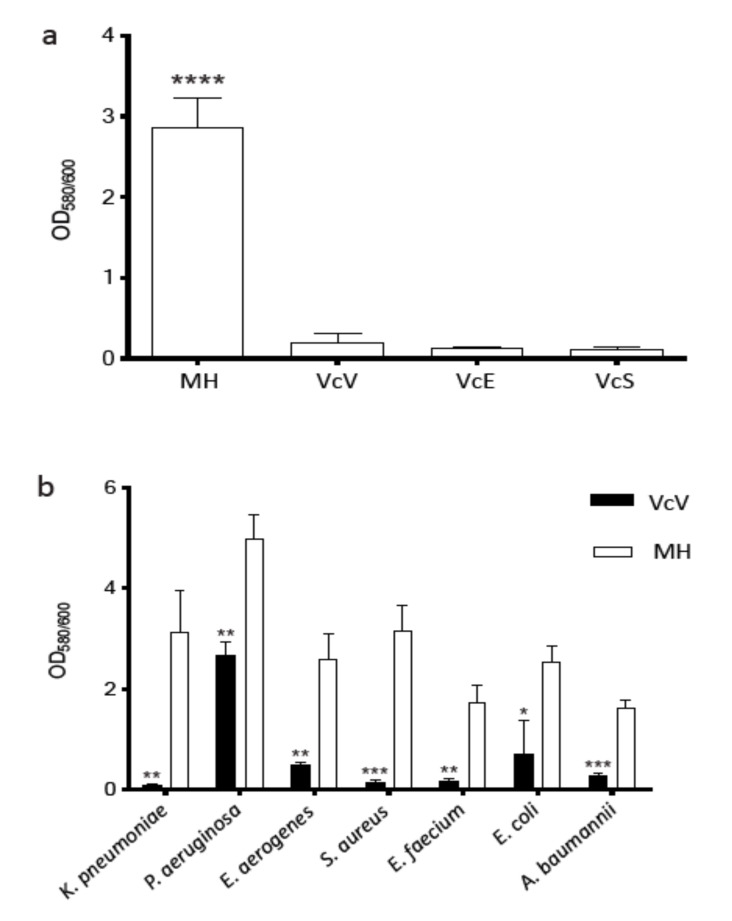
Quantification of biofilm formation by crystal violet staining: (**a**) *K. pneumoniae* clinical strain is denoted as statistically significant and (**b**) other bacterial strains. MH: absence of blueberry extract, presence of VcV (26.2 μg mL^−1^), VcS (10.2 μg mL^−1^), and VcE (10.1 μg mL^−1^). Six independent biological replicates were analyzed. The statistical significance between the VcV group and the respective MH control is indicated (* *p* ≤ 0.05, ** *p* ≤ 0.01, *** *p* ≤ 0.001, **** *p* < 0.0001).

The effect of the three phenolic extracts on the biofilm produced by *K. pneumoniae* was also investigated. The results are shown in Figure 3. A crystal violet assay was used to quantify the biofilm production and it revealed that all extracts (VcV, VcE, and VcS) significantly reduced the biofilm formation (*p* < 0.0001) in *K. pneumoniae* (Figure 3a). Significant differences among the different extracts in terms of efficacy were not detected.

Considering that the VcV extract contains a higher amount of bioactive polyphenols, its antibiofilm activity was tested in the same MDR pathogens previously selected for the bactericidal assays. Figure 3b shows that the VcV extracts remarkably inhibit the biofilm formation in all the clinical strains tested. It has been also observed that the VcV extract (bioactive polyphenols: 26.2 μg mL^−1^) inhibited the biofilm formation differently, depending on the bacterial species. The greatest effect was found in *K. pneumoniae*, in which the biofilm produced was reduced by 97.47%. In *S. aureus* this reduction was 95.1%, followed by *E. faecium* with 90.16%, and *A. baumannii*, *E. aerogenes*, *E. coli*, and *P. aeruginosa* with 82.45%, 81.36%, 71.64%, and 46.42%, respectively (Figure 3b).

### 2.3. Analytical Characterization of Pilot-Scale Blueberry Extracts

Once it was demonstrated that the blueberry extracts obtained by MSATs have a polyphenolic profile that enables them to be used as an antibiofilm and a disinfectant (bacteriostatic and bactericide activities), its production on a pilot scale was proposed as a proof-of-concept demonstration. A total of 7 L of saline extract were obtained from 5 kg of bulk blueberries of the Bluecrop variety (VcB). The selection of the variety was conditioned by the availability of blueberries in bulk, once it was proven that the extracts of all the varieties previously tested presented good antibacterial behavior. It must be taken into account that extraction on an industrial scale is considered, either by taking advantage of the byproducts from the blueberry processing industry, or as an alternative way of managing whole fruits that do not have a “premium” quality; that is why a raw material with these characteristics was acquired, seeking to give it an added value.

**Figure 4:**
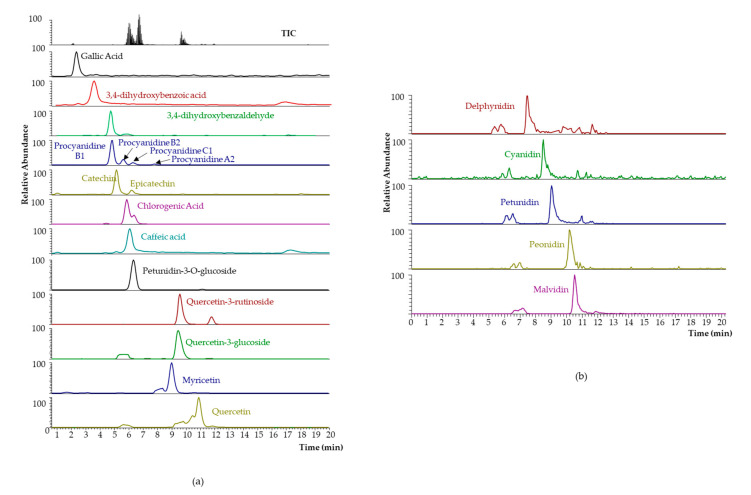
LC–MS/MS extracted chromatograms (see specific MS/MS transitions in Table S1) for the proof-of-concept VcB extract obtained at a pilot scale: (**a**) non-flavanoids, flavan-3-ols, procyanidins, flavonols, and anthocyanins; and (**b**) free anthocyanidins released by the alkaline hydrolysis of the extract (see concentrations in Table 2).

The obtained pilot-scale blueberry extract presented a red color, somehow between burgundy and carmine, with a maximum absorbance in the wavelength range of 510–525 nm, but also with significant absorbance of around 400 nm and 750 nm. Its chemical characterization was performed, giving a TPC value of 1455 ± 288 mg GAE L^−1^. Individual polyphenolic quantification and the obtained chromatograms are depicted in Table 2 and Figure 4, respectively. Its composition was compared with a blueberry extract obtained by MSATs with the same raw material (VcB) (TPC = 512 ± 12 mg GAE L^−1^). The phenolic composition is also shown in Table 2. Qualitatively, the differential characteristics of the VcB variety are the presence of protocatechuic acid, procyanidin C1 (in a very significant amount), and myricetin, and the absence of epicatechin-gallate.

## 3. Discussion

A countless number of publications about the antimicrobial activity of phytochemicals, in general, and polyphenols in particular, are reported [16,33,34,35]. The relations between such activity and specific chemical families, or even individual chemical compounds, have been described therein, including structure–activity relationships [16,34]. In the particular case of berry extracts, the antibacterial activity has been mainly attributed to proanthocyanidins and anthocyanins, but also important effects of flavan-3-ols, flavonols, and phenolic acids have been attributed [33]. The starting hypothesis is that there is a direct relationship between the antimicrobial activity of the extract and its polyphenolic composition. There are also studies reinforcing this argument, that individual phytochemicals do not work as effectively as heterogeneous extracts [35].

The comparative analysis of the polyphenolic composition of the obtained *V. corymbosum* extracts with those reported in the literature is complicated, as this composition depends on many factors related to the cultivar itself and to the extraction method, especially linked to the extraction solvent. Since, in this work, no organic solvent is used that can imply a low extraction efficiency for some of the less polar polyphenols, the representative compounds of all the polyphenolic families of blueberries have been obtained in enough quantity to achieve a potent bioactive extract, with the additional advantage of direct application for the control of target pathogens without toxicity restrictions.

The distribution of the different flavonoid families in the analyzed cultivars indicates a clear predominance of anthocyani(di)ns, with a similar proportion in the VcV and VcS varieties compared to the VcE variety. However, the concentration is much higher in the VcV variety, being 2.5 times that of VcS and more than 6 times that of VcE. Flavonols are the second most abundant group of flavonoids, although an order of magnitude lower than the anthocyanin family. The Ventura variety triples the quantity of the other two, which show equivalent contents. The concentration range of procyanidins varies between cutivars, being B1 and B2 more abundant in Ventura, and A2 in the Emerald cultivar. These results are in concordance with those reporting similar amounts of total procyanidins in the blueberries of other varieties, with higher values determined in cultivated varieties than in the wild ones [25]. The levels of flavan-3-ols were similar for VcV and VcS, duplicating those of the VcE variety. The total content of bioactive polyphenols determined by LC–MS/MS is much higher in VcV, followed by VcS, and then by VcE. However, from a qualitative point of view, the profile of the three liquid extracts is equivalent.

Analyzing the individual concentrations of the polyphenols detected, petunidin-3-O-glucoside stands out; it was by far the most abundant compound detected in the extracts, although the quantities differed greatly between the three varieties. The Ventura variety presented the highest total load of anthocyanidins, followed by VcS and VcE. Malvidin was the major anthocyanidin in all the cultivars studied, followed by the flavonols quercetine and isoquercetine, highlighting its content, once again, in the VcV variety. Finally, chlorogenic acid was the main non-flavonoid polyphenol, showing equivalent quantities in the three studied varieties.

All the tested extracts showed a clear antimicrobial effect, which suggests that the qualitative profile of an extract plays a key role in its antimicrobial behavior. Moreover, a relationship between the concentration of bioactive polyphenols in the extract and its efficacy as antimicrobial was found. The VcV extract, which contained the highest amount of bioactive polyphenols, was the most active. Extensive research has demonstrated the antimicrobial properties of many berry extracts, attributing their inhibitory effect to the high content of phenolic constituents [12,13,14]. Thus, the efficacy of the extract is associated to the polyphenolic content, which can substantially vary depending on the extraction method, the solvent, or the growth conditions. However, most of the efforts were focused on investigating the antimicrobial effect against foodborne- and urinary tract infection-related pathogens [12,36]. The growth inhibitory effect of the VcV extract was observed in all the pathogens tested. Despite the slight differences in the growth patterns shown by the different bacterial strains, none of them were able to grow in the presence of the blueberry extract. The VcV extract inhibited the growth of all of them, despite being different species and having a different mechanism of resistance. This can be explained by the fact that the inhibitory effects of most polyphenols are concentration-dependent [12,13,33], but also because polyphenols themselves have multiple mechanisms of action, which explains the bacteriostatic or bactericidal effects observed against a wide array of bacteria [16].

Therefore, the obtained results of this work are in concordance with those previously reported. If the concentration is high enough (e.g., over 100 μg mL^−1^), the blueberry extract is active against the seven concerning multidrug-resistant pathogens tested. Moreover, the mechanism of action of these berry extracts is apparently able to overcome the classical mechanism of resistance, as they are effective against both susceptible and multidrug-resistant strains. Although, the vast majority of polyphenolic-rich extracts have minimum inhibitory concentrations (MICs) that are higher than antibiotics and their use as antimicrobial in monotherapy is not appropriate; the combined therapy with antibiotics can not only improve their efficacy, but can also reduce the emergence of antibiotic resistance. N. Sila et al. [16] published an in-depth study on plant-derived natural products inhibiting biofilm formation. Many of the polyphenols found in the composition of the blueberry extracts obtained in the present work are related to the inhibition of biofilm formation in certain bacterial species. Different phytochemicals showed antibiofilm activities against the corresponding bacteria: *Pseudomonas aeruginosa* (catechin, epigallocatechin, quercetin, gallic acid, and chlorogenic acid); *Klebsiella* pneumonia (malvidine); *Escherichia coli* (quercetin, rutin, myricetin, and gallic acid); *Staphylococcus aureus* (quercetin, myricetin, gallic acid, protocatechuic acid, caffeic acid, and chlorogenic acid). Furthermore, chlorogenic acid also acts against biofilms formed by the bacteria of the genera *Acinetobacter* and *Enterobacter*. Some of these studies [16] are based on the activity of individual chemical compounds, while others refer to the multicomponent extracts of natural origin, such as those obtained in this study. In certain cases, evidence that individual polyphenols of a synthetic origin has little to no antibacterial activity, while how the synergy between polyphenols making up natural extracts triggers their antimicrobial potential has been shown [19]. The synergy between all the secondary metabolites synthesized by a plant makes the extracts of natural origin a powerful broad-spectrum antimicrobial agent. For this reason, the blueberry saline extracts studied are effective against all nosocomial pathogens tested, making them a potential product for direct use in the disinfection of biotic and abiotic surfaces. The bacteria in the biofilm form contribute to the persistent infections associated with implanted medical devices and, although indwelling medical devices are often primarily colonized by single bacterial species, after a short time, a multispecies consortium quickly develops [16]. Thus, the best strategy involves a multi-component extract that is active against multiple biofilm-forming bacterial species.

Considering its application as a disinfectant product on biotic and abiotic surfaces, this approach can avoid the need of combining the extract with synthetic antibiotics, reducing their indiscriminate use, thus reducing the impact of MDR bacteria. The presence of different polyphenols in the multicomponent extract probably leads to the combination of different mechanisms of action acting on different virulence factors, such as the capacity of the flavonols to penetrate cell phospholipid membranes due to their hydrophobicity [37], or the destabilization and permeabilization of the cell membrane, inhibition of extracellular microbial enzymes, and other mechanisms attached to proanthocyanidins [38] and to some non-flavonoids, such as chlorogenic acid [39].

Interestingly, the scaling up of the extraction process increases the bioactive polyphenols content up to three times. This effect was already observed for white grape marc [31], and can be attributed to the fact that the amount of starting material is also much higher. The plant phenolics most affected by this increment were anthocyani(di)ns. Flavan-3-ols also increased, but their content remained relatively small in the final extract, compared to the other families. The contents of procyanidins and flavonols were about the same, in general terms. The increase in non-flavonoids was almost entirely due to chlorogenic acid.

Therefore, from the above results, it has been demonstrated that the obtained proof-of-concept extract presents antibiofilm, growth inhibitory effect, and bacteriostatic activity against the nosocomial pathogens assessed. It is also expected to show effective bactericidal activity, as its bioactive polyphenols content is close to that of VcV, more than double that of the VcS, and more than four times that of VcE. It has been proven that an active extract rich in polyphenols can be obtained from bulk blueberries, using a technology that operates in mild conditions, at room temperature and atmospheric pressure, and in a saline medium compatible with the direct treatment of biotic or abiotic surfaces.

## 4. Materials and Methods

### 4.1. Reagents, Solvents, and Materials

The target polyphenols, their identification CAS numbers, suppliers, retention times, and MS/MS transitions are summarized in Table S1.

Washed sea sand (200–300 μm), glass wool, and anhydrous sodium sulphate (Na_2_SO_4_) were obtained from Scharlau (Barcelona, Spain). The extraction solvent used was distilled water (Millipore, Bedford, MA, USA) with dissolved salts. Methanol and water (MS grade) were provided by Scharlau, and formic acid (98–100%) by Merck (Darmstadt, Germany). The gallic acid and Folin–Ciocalteu phenol reagent were obtained from Sigma-Aldrich (Steinheim, Germany).

Individual standard stock solutions (1000–10,000 μg mL^−1^) were prepared in methanol. Working solutions were weekly prepared by dilution in water. Stock and working solutions were stored in a freezer at −20 °C and protected from light. All solvents and reagents were of analytical grade.

External calibration was used for the quantification of polyphenols. Linearity was evaluated in a wide range of concentrations from 0.01–10 μg mL^−1^ (8 levels and 3 replicates per level) for polyphenols and between 1–50 μg mL^−1^ (6 replicates and 3 replicates per level) for anthocyanins, employing standard solutions prepared in water:methanol (95:5 v/v). The obtained coefficients of determination (R^2^) were, in all cases, higher than 0.9900. The instrumental detection limits (IDLs) were calculated as the compound concentration giving a signal-to-noise ratio of 3 (S/N = 3), since none of the target compounds were detected in the mobile phase and solvent blanks. The obtained IDLs for the compounds quantified in the blueberry extracts are shown in Table S2.

### 4.2. Samples and Sample Pre-Treatment

Three different high-bush cultivars of blueberries (*Vaccinium corymbosum* from the *Ericaceae* family) were used in this study for medium-scale extractions and will be referred to as VcV (Ventura), VcS (Star), and VcE (Emerald). All of them, properly labeled with their genus and species, were purchased fresh from local fruit shops (Santiago de Compostela, Spain), and immediately frozen. The different varieties of blueberries have different cold requirements and different phenological characteristics. The cultivars tested in this study were all low chill, with berry sizes that were medium (VcS), large (VcV), and very large (VcE). The pilot scale proof-of-concept experiment was carried out with blueberries from a bulk of the Bluecrop cultivar (VcB), the industry’s most widely planted variety and the one recommended to be processed.

Frozen samples were crushed before each extraction. This allowed the amount of polyphenols obtained in each extraction to be maximized, enriching the extracts and increasing their added value. In the crushing stage, the meticulous crushing of the skin was key to efficiently extracting all the anthocyanins. For the independent experiments on peel and pulp, these were manually separated.

The water content of each raw material was calculated by the classical method of bringing a known quantity of sample to a constant weight in an oven at 105 °C. the percentages of humidity were 84.6% (VcV), 86.3% (VcE), 90.7% (VcS), and 90.1% (VcB).

### 4.3. Extraction Systems at Medium and Pilot Scale

Blueberry extracts were first obtained at medium scale, ambient temperature, and atmospheric pressure by a system consisting in an adapted tubular glass column (20 cm height × 5 cm diameter) containing layers of glass wool and sand at both ends, acting as an in-situ clean-up filter (the bottom one) and as an extraction solvent diffuser (the upper one) [32]. A total of 20 g of raw material was extracted to obtain ready-to-use extract volumes over 100 mL. The frozen blueberries were dispersed with sand and the drying agent Na_2_SO_4_ (ratio 1:2:2 *w*/*w*), by grounding in a glass mortar until a homogeneous mixture was obtained. Then, it was loaded onto the column, and the elution solvent (saline water, SW) was poured. The elution solvent is an isotonic aqueous solution formulated in such a way that its salinity is equal to that of the human body, with its main components being sodium, potassium, calcium, magnesium, and chloride (modified Hanks’ balanced salt solution, Merck (Darmstadt, Germany)). The freeze drying of the extracts was carried out, when necessary, in a Telstar freeze dryer Cryodos model with a final condenser temperature of −55 °C.

The extraction at pilot scale was performed in a stainless steel column designed in such a way that the geometry maintained the proportions of the system used at medium scale. The column, with a diameter of 20 cm and a total height of 70 cm (useful height 65 cm), has two valves, one in the upper part in which the extraction solvent is loaded and another in the lower part that facilitates obtaining the eluate. The column is mounted on an iron support structure that allows it to rotate by 180° to facilitate loading and unloading. Five kilograms of bulk blueberries were used as raw material and dispersed with sand. A total of 7 L of extract was obtained in the proof-of-concept demonstration using 5 L of SW. The additional volume comes from the fruit juice of the blueberries itself.

### 4.4. Analytical Characterization of the Blueberry Extracts

The TPC of the *Vaccinium corymbosum* extracts were determined by the Folin–Ciocalteu spectrophotometric method [40]. The TPC was quantified employing a calibration curve ranging from 3 to 20 mg L^−1^ (R^2^ = 0.9970) prepared with the gallic acid standards solutions and expressed as mg equivalents of gallic acid (GAE) per L of liquid extract (mg GAE L^−1^). The visible spectrum of the proof-of-concept extract was also measured. In both cases, a UV mini 1240 spectrophotometer (Shimazdu, Kyoto, Japan) was employed.

The individual polyphenols present in the blueberry extracts were identified and quantified by LC–MS/MS analysis, employing a thermo scientific (San José, CA, USA) instrument based on a TSQ Quantum Ultra^TM^ triple quadrupole mass spectrometer equipped with a HESI-II (heated electrospray ionization), and an Accela Open autosampler with a 20 µL loop. Optimal instrumental conditions were adapted from Celeiro et al. [41]. The chromatographic separation was achieved on a Kinetex C18 column (100 × 2.1 mm, 2.6 µm, 100 Å), obtained from Phenomenex (Torrance, CA, USA). The temperature of the column was set at 50 °C. The mobile phase consisted of water (A) and methanol (B), both with 0.1% formic acid. The eluted gradient started with 5% of B (held at 3.5 min), it was increased to 90% of B in 11 min and kept constant for 3 min. Finally, initial conditions were achieved in 6 min. The injection volume was 10 µL and the mobile phase flow rate was 0.2 mL min^−1^. The total run time for each injection was 20 min. The MS/MS parameters for all the compounds studied were optimized by individual direct infusion and the most abundant collision-induced fragments were considered for quantification (Table S1). Other HESI source parameters were the spray voltage: 3000 V, vaporizer temperature: 350 °C, sheath gas pressure: 35 au (arbitrary units), and ion sweep and auxiliar gas pressure: 0 and 10 au, respectively, and the capillary temperature: 320 °C. The mass spectrometer and the HESI source were working simultaneously in the positive and negative mode, monitoring two or three MS/MS transitions for each compound (see Table S1) for an unequivocal identification and quantification of the target compounds. The system was operated by Xcalibur 2.2 and Trace Finder^TM^ 3.1 software.

### 4.5. Evaluation of the Antimicrobial Activity

#### 4.5.1. Bacterial Strains and Culture Conditions

A total of 7 nosocomial pathogens (5 Gram-negative and 2 Gram-positive) were used in this study. These clinical strains were selected taking into consideration the list published by the WHO, reporting the antibiotic-resistant pathogens for which new and effective antibiotics are urgently needed [2]. The selected pathogens include *Acinetobacter baumannii*, *Pseudomonas aeruginosa*, *Klebsiella pneumoniae*, *Escherichia coli*, and *Enterobater aerogenes*, all of them resistant to carbapenems, and vancomycin-resistant *Enterococcus faecium* and methicillin-resistant *Staphylococcus aureus*. The three *Enterobacteriaceae* (*Klebsiella pneumoniae, Escherichia coli*, and *Enterobater aerogenes*) are OXA-48 and CTX-M-15 producers.

Strains were grown in *Luria Bertani* (LB) broth (10 g L^−1^ tryptone, 5 g L^−1^ yeast extract, and 10 g L^−1^ NaCl) or on LB agar (LB broth supplemented with 20 g L^−1^ of agar). The strains were routinely grown at 37 °C with shaking and stored at −80 °C in LB broth with 20% glycerol.

#### 4.5.2. Killing Curves Assay

Time–kill analysis was used to measure the in vitro bactericidal activity of the VcV, VcE, and VcS polyphenolic extracts. The clinical strain was grown on LB broth overnight at 37 °C under agitation. The overnight culture was 1:100 diluted in 5 mL of Mueller–Hinton (MH) medium, and MH supplemented with phenolic extracts obtaining a bacterial density of ca. 5 × 10^6^ colony-forming units (CFUs)/mL. The bacterial cultures were incubated at 37 °C under constant shaking at 180 rpm for 24 h. The number of CFUs was determined every hour during the first 12 h and a final measurement at 24 h. Dilutions of the culture were plated onto LB agar and incubated at 37 °C for 24 h. Three independent biological replicates were performed.

#### 4.5.3. Growth Curves Assay

The growth rate of the clinical strains was measured to determine the inhibitory effect of the VcV polyphenolic extract. Assays were performed in an MH medium (control condition) and in MH supplemented with polyphenolic extract. The clinical strains were grown on LB broth overnight at 37 °C with shaking. The overnight cultures were 1:100 diluted and incubated in polystyrene 48-well flat bottom microtiter plates (Corning^®^ Costar^®^ TC-Treated Multiple Well Plates, Sigma Aldrich, Steinheim, Germany) containing 250 mL of culture. Microtiter plates were then incubated at 37 °C with constant shaking at 180 rpm and the growth was monitored using an Epoch 2 microplate spectrophotometer (BioTek Instruments, Inc., Santa Clara, CA, USA). The OD_600_ values were recorded every 10 min, with hourly data being plotted on the graph. Three independent biological replicates were performed for each strain.

#### 4.5.4. Statistical Analysis

The statistical analysis in killing curve and growth curve assays were evaluated through the two-stage linear step-up procedure of Benjamini, Krieger, and Yekutieli, with Q = 1%. Each row was individually analyzed, without assuming a consistent SD.

#### 4.5.5. Quantitative Biofilm Assay

Biofilm formation was quantified following a previously described procedure [18] with some modifications. The clinical strains were grown on LB broth overnight at 37 °C with shaking. The overnight culture of each strain was 1:100 diluted in Mueller–Hinton (MH) medium, and MH was supplemented with phenolic extracts (VcV, VcE, or VcS) and independently inoculated in polystyrene 48-well flat bottom microtiter plates (Corning^®^ Costar^®^ TC-Treated Multiple Well Plates). The plates were incubated at 37 °C for 24 h under static conditions and the biofilm formation was visualized by staining with 0.1% crystal violet assay. Bacterial growth was measured at OD_600_ to estimate the total cell biomass. Biofilm formation was quantified (OD_580_) after solubilization with 30% acetic acid. The amount of biofilm formed was determined as the OD_580_/OD_600_ ratio to prevent variations due to differences in bacterial growth. Six independent biological replicates were performed. ANOVA and student’s t-tests were used to statistically validate the experimental data.

## 5. Conclusions

Extracts obtained from three *Vaccinium corymbosum* varieties, Ventura (VcV), Star (VcS), and Esmerald (VcE) showed a clear antimicrobial effect. The results revealed that a relationship exists between the concentration of bioactive polyphenols and the efficacy of the extract as an antimicrobial agent. It has been further demonstrated that a multicomponent polyphenolic extract of these characteristics can be obtained on an industrial scale, by maintaining a green and sustainable extraction procedure that respects the principles of green chemistry, with an excellent yield, and from fruit processing wastes or low-quality blueberries unsuitable for direct consumption. The obtained results suggest that this type of extract can potentially act as a new therapeutic alternative and/or antibiotic adjuvant in order to control infections caused by these pathogens.

## Figures and Tables

**Table 1:** Polyphenolic composition of saline blueberry extracts obtained by MSATs from three different commercial varieties.

Plant Polyphenols Group	Blueberry cultivar	VcV	VcE	VcS
	Compound Name	Concentration (µg g^−1^ dw)		
**Benzoic acids**	**Gallic acid**	2.4 ± 0.3	2.5 ± 0.6	1.9 ± 0.5
Hydroxycinnamic acids	Caffeic acid	2.6 ± 0.5	2.5 ± 0.4	9.6 ± 1.1
	Chlorogenic acid	708 ± 22	154 ± 40	781 ± 72
Phenolic aldehydes	Protocatechualdehyde	2.8 ± 0.2	1.6 ± 0.3	4.9 ± 0.4
**∑ Non-Flavanoids**		**716**	**161**	**797**
Flavan-3-ols	Catechin	2.7 ± 0.9	0.27 ± 0.08	nd
	Epicatechin	0.5 ± 0.2	nd	0.2 ± 0.1
	Epicatechin-gallate	5.8 ± 0.9	4.6 ± 2.0	9.9 ± 6.4
**∑ Flavan-3-ols**		**9**	**5**	**10**
Flavan-3-ols oligomeric derivatives	Procyanidine A2	0.3 ± 0.1	7.6 ± 2.3	0.5 ± 0.2
	Procyanidine B1	3.5 ± 0.3	0.17 ± 0.09	0.20 ± 0.03
	Procyanidine B2	3.5 ± 1.4	0.5 ± 0.3	0.5 ± 0.2
**∑ Procyanidines**		**7.3**	**8.3**	**1.2**
Flavonols	Quercetin	760 ± 61	336 ± 49	10.6 ± 5.2
	Isoquercetin	1300 ± 146	348 ± 40	592 ± 108
	Rutin	75 ± 13	7.7 ± 1.4	121 ± 24
**∑ Flavonols**		**2135**	**692**	**724**
Anthocyanidins ^1^	Delphinidin	153 ± 24	nd	nd
	Cyanidin	269 ± 47	35 ± 7	84 ± 5
	Petunidin	812 ± 24	18.5 ± 2.4	122 ± 34
	Peonidin	121 ± 33	17 ± 5	114 ± 11
	Malvidin	2895 ± 861	339 ± 36	2113 ± 251
Anthocyanins	Petunidin-3-*O*-glucoside	18,485 ± 468	3134 ± 283	6167 ± 363
**∑ Anthocyani(di)ns**		**22,735**	**3544**	**8599**
**∑ Flavanoids**		**24,886**	**4248**	**9334**
**∑ Bioactive Polyphenols**		**25,602**	**4409**	**10,133**

^1^ Concentrations determined by the alkaline hydrolysis of the extract to free anthocyanins from their corresponding sugar derivatives.

**Table 2:** Polyphenolic content of the saline blueberry extract (VcB) obtained by MSATs and at a pilot scale.

Plant Polyphenols Group	Compound Name	MSATs	Pilot Scale
		Concentration (µg g^−1^ dw)	
**Benzoic acids**	**Gallic acid**	2.6 ± 0.2	3.96 ± 0.07
	Protocatechuic acid	0.61 ± 0.08	2.8 ± 0.4
Hydroxycinnamic acids	Caffeic acid	4.45 ± 0.09	5.2 ± 0.1
	Chlorogenic acid	475 ± 4	978 ± 3
Phenolic aldehydes	Protocatechualdehyde	1.3 ± 0.2	1.0 ± 0.2
**∑ Non-Flavanoids**		**484**	**991**
Flavan-3-ols	Catechin	7.47 ± 0.06	30 ± 5
	Epicatechin	1.7 ± 0.3	7 ± 2
**∑ Flavan-3-ols**		**9**	**37**
Flavan-3-ols oligomeric derivatives	Procyanidine A2	-	0.513 ± 0.03
	Procyanidine B1	20.8 ± 0.5	50 ± 2
	Procyanidine B2	18.6 ± 0.1	42 ± 4
	Procyanidine C1	302 ± 43	316 ± 39
**∑ Procyanidines**		**341**	**408**
Flavonols	Quercetin	17 ± 1	27 ± 4
	Isoquercetin	791 ± 25	846 ± 31
	Rutin	128 ± 4	130 ± 1
	Myricetin	8.9 ± 1.6	19 ± 4
**∑ Flavonols**		**945**	**1022**
Anthocyanidins ^1^	Delphinidin	150 ± 17	1983 ± 87
	Cyanidin	75 ± 13	248 ± 10
	Petunidin	149 ± 29	1239 ± 16
	Peonidin	17 ± 4	60 ± 4
	Malvidin	1220 ± 67	7182 ± 68
Anthocyanins	Petunidin-3-*O*-glucoside	3565 ± 35	7664 ± 155
**∑ Anthocyani(di)ns**		**5176**	**18,376**
**∑ Flavanoids**		**6471**	**19,843**
**∑ Bioactive Polyphenols**		**6955**	**20,834**

^1^ Concentrations determined by alkaline hydrolysis of the extract to free the anthocyanins from their corresponding sugar derivatives.

## Data Availability

Data are available within the present article and Supplementary Materials.

## Supplementary Materials

The following are available online at https://www.mdpi.com/article/10.3390/plants10122801/s1, Table S1: studied compounds. Ionization mode, CAS number, retention time, and MS/MS transitions; Table S2: LC–MS/MS performance for the 22 polyphenols identified in the blueberry extracts.
