# Supplementary material for: Multicomponent Polyphenolic Extracts from Vaccinium corymbosum at Lab and Pilot Scale. Characterization and Effectivity against Nosocomial Pathogens
Source: Plants (Basel). 2021 Dec 17;10(12):2801. doi: 10.3390/plants10122801 (PMC8708234; doi:10.3390/plants10122801)
Supplement: Supplementary file 1 [file plants-10-02801-s001.zip › plants-1489867-supplementary.pdf]

Supplementary material

Multicomponent polyphenolic extracts from *Vaccinium corymbosum* at lab and pilot scale. Characterization and effectivity against nosocomial pathogens

Eva Gato [1], Astrid Perez[1*], Alicja Rosalowska [2], Maria Celeiro [3], German Bou [1] and Marta Lores [2,*]

[1] Departamento de Microbiología, Complejo Hospitalario Universitario A Coruña (CHUAC), Instituto de Investigación Biomédica A Coruña (INIBIC), Universidad de A Coruña (UDC), A Coruña (Spain); eva.gato.corral@sergas.es; astrid.perez.gomez@gmail.com; german.bou.arevalolsergas.es

[2] LIDSA, Department of Analytical Chemistry, Nutrition and Food Science, Universidade de Santiago de Compostela, E-15782 Santiago de Compostela, Spain; marta.lores@usc.es; ally.rosalowska@gmail.com

[3] CRETUS, Department of Analytical Chemistry, Nutrition and Food Science, Universidade de Santiago de Compostela, E-15782 Santiago de Compostela, Spain; maria.celeiro.montero@usc.es

* Correspondence: astrid.perez.gomez@gmail.com (A.P.); marta.lores@usc.es (M.L.)

Table S1. Studied compounds. CAS number, ionization mode, retention time,MS/MS transitions and suppliers.

Compound	CAS	Ionization mode	Retention time (min)	MS/MS transitions (Collision energy, eV)[a]
Gallic acid [b]	149-91-7	-	2.25	<u>169.02 → 125.04 (17)</u><br>169.02 → 153.1 (15)
2,4,6-trihydrobenzoic acid [b]	487-70-7	+	3.23	<u>168.98 → 150.99 (17)</u><br>168.98 → 83.02 (23)<br>168.98 → 107.02 (22)
2,4-dihydroxybenzoic acid [b]	89-86-1	+	3.69	<u>153.00 → 109.05 (16)</u><br>153.00 → 65.09 (19)<br>153.00 → 67.07 (23)
3,4-dihydroxybenzoic acid [b]	99-50-3	+	3.70	<u>152.98 → 109.04 (17)</u><br>152.98 → 91.04 (28)<br>152.98 → 108.03 (26)
Caftaric Acid [b]	67879-58-7	-	4.21	<u>310.96→ 178.97 (17)</u><br>310.96 → 148.96 (14)
3,4-dihydroxybenzaldehyde [b]	139-85-5	+	4.55	<u>137.07 → 136.11 (21)</u><br>137.07 → 91.09 (24)<br>137.07 → 92.13 (25)
2,5-dihydroxybenzoic acid [b]	490-79-9	+	4.74	<u>152.96 → 108.00 (24)</u><br>152.96 → 81.02 (21)<br>152.96 → 109.01 (16)
Procyanidin B1 [c]	20315-25-7	-	4.76	<u>577.03 → 407.06 (26)</u><br>577.03 → 288.93 (25)<br>577.03 → 424.97 (26)

2,6-dihydroxybenzoic acid [b]	303-07-1	+	4.86	153.00 → 109.05 (17)<br>153.00 → 65.09 (21)<br>153.00 → 135.02 (16)
3,5-dihydroxybenzoic acid [b]	99-10-5	+	4.90	152.97 → 109.01 (15)<br>152.97 → 65.06 (16)<br>152.97 → 67.05 (20)
Catechin [b]	225937-10-0	+	5.02	289.00 → 245.02 (17)<br>289.00 → 203.11 (22)
Procyanidin B2 [b]	29106-49-8	-	5.50	577.03 → 407.06 (26)<br>577.03 → 288.93 (25)<br>577.03 → 424.97 (26)
2,5-dihydroxybenzaldehyde [b]	1194-98-5	+	5.50	136.99 → 108.02 (21)<br>136.99 → 81.08 (18)<br>136.99 → 109.04 (14)
4-hydroxybenzaldehyde [b]	123-08-0	+	5.62	122.97 → 95.05 (13)<br>122.97 → 51.10 (36)<br>122.97 → 77.05 (20)
3-hydroxybenzaldehyde [b]	90-02-8	+	5.63	121.02 → 93.05 (20)<br>121.02 → 92.05 (23)<br>121.02 → 120.04 (19)
3-hydroxybenzoic acid [b]	99-06-9	+	5.68	136.94 → 93.07 (14)<br>136.94 → 65.09 (24)<br>136.94 → 91.01 (32)
Chlorogenic acid [b]	327-97-9	+	5.77	353.00 → 191.07 (22)<br>353.00 → 85.09 (43)<br>353.00 → 93.07 (45)
3,4-dimetoxybenzoic acid [d]	93-07-2	+	5.92	182.96 → 137.08 (6)<br>182.96 → 106.99 (22)
Caffeic Acid [b]	331-39-5	-	5.92	178.98 → 135.03 (19)<br>178.98 → 134.01 (28)
Epigallocatechingallate [e]	989-51-5	+	6.00	457.15 → 169.05 (21)<br>457.15 → 125.09 (42)<br>457.15 → 305.09 (21)
Procyanidin C1 [b]	37064-30-5	-	6.01	577.03 → 288.93 (25)<br>577.03 → 407.06 (26)<br>577.03 → 424.97 (26)
Epicatechin [b]	490-46-0	+	6.11	289.00 → 245.02 (17)<br>289.00 → 203.11 (22)
Petunidin-3-O-glucoside [b]	6988-81-4	+	6.49	303.02 → 115.08 (50)<br>303.02 → 173.07 (33)<br>303.02 → 257.07 (27)
Procyanidin A1 [d]	103883-03-0	-	6.50	577.03 → 288.93 (25)<br>577.03 → 407.06 (26)<br>577.03 → 424.97 (26)
Gallocatechingallate [b]	4233-96-9	+	6.59	457.15 → 169.05 (21)<br>457.15 → 125.09 (42)<br>457.15 → 305.09 (21)
Trans-4-hydroxycinnamic acid [b]	501-98-4	+	7.12	163.02 → 119.07 (18)<br>163.02→ 93.07 (37)<br>163.02 → 117.05 (38)
Epicatechingallate [b]	1257-08-5	+	7.13	441.13 → 289.13 (30)<br>441.13 → 125.08 (42)<br>441.13 → 169.05 (24)
7-hydroxycoumarin [d]	779-27-1	+	7.14	162.99 → 107.04 (22)<br>162.99 → 77.05 (34)<br>162.99 → 91.05 (20)

Catechingallate [b]	130405-40-2	+	7.17	441.13 → 289.13 (20)<br>441.13 → 125.08 (42)<br>441.13 → 169.05 (24)
Procyanidin A2 [b]	41743-41-3	-	7.35	577.09 → 287.00 (32)<br>577.09 → 136.98 (62)<br>577.09 → 425.08 (13)
Orientin [c]	28608-75-5	-	7.64	447.16 → 327.14 (23)<br>447.16 → 357.16 (22)
Delphynidin [c,f]	528-53-0	+	7.81	303.02 → 229.07 (32)<br>303.02 → 115.08 (50)<br>303.02 → 173.07 (33)
3,4-dimethoxybenzaldehyde [b]	120-14-9	+	8.23	167.01 → 139.05 (13)<br>167.01 → 108.05 (21)<br>167.01 → 124.03 (18)
Cyanidin [b,f]	528-58-5	+	8.87	287.02 → 128.10 (46)<br>287.02 → 109.06 (39)<br>287.02 → 115.08 (45)
4-methoxybenzaldehyde [d]	123-11-5	+	9.05	136.97 → 109.05 (12)<br>136.97 → 77.05 (23)<br>136.97 → 94.04 (18)
Quercetin-3-glucuronide [b]	22688-79-5	+	9.21	479.09 → 461.50 (14)<br>479.09 → 302.96 (18)
Quercetin-3-rutinoside [b]	207671-50-9	-	9.39	609.18 → 270.92 (96)<br>609.18 → 178.87 (44)<br>609.18 → 300.01 (37)
Quercetin-3-glucoside [b]	482-35-9	+	9.41	465.07 → 256.90 (41)<br>465.07 → 302.97 (14)
Petunidin [b,f]	1429-30-7	+	9.43	317.03 → 302.05 (25)<br>317.03 → 217.03 (40)<br>317.03 → 245.03 (36)
Pelargonidin [c,f]	134-04-3	+	10.09	271.05 → 121.10 (33)<br>271.05 → 115.14 (45)<br>271.05 → 141.15 (34)
Myricetin [d]	529-44-2	+	10.30	319.00 → 153.02 (31)<br>319.00 → 217.06 (31)<br>319.00 → 245.06 (27)
Peonidin [c,f]	134-01-0	+	10.60	301.03 → 286.08 (24)<br>301.03 → 201.07 (38)<br>301.03 → 229.07 (38)
Malvidin [c,f]	643-84-5	+	10.90	331.11 → 315.14 (30)<br>331.11 → 242.13 (31)<br>331.11 → 287.14 (30)
3,4,5-trimethoxycinnamic acid [b]	90-50-6	+	11.08	239.03 → 221.04 (11)<br>239.03 → 162.99 (27)<br>239.03 → 190.01 (19)
3,5-dimethoxybenzaldehyde [b]	7311-34-4	+	11.23	167.15 → 124.03 (17)<br>167.15 → 77.05 (26)
Quercetin [b]	117-39-5	+	11.70	303.09 → 229.10 (28)<br>303.09 → 153.04 (33)
Kaempferol [b]	520-18-3	-	12.27	285.07 → 184.91 (30)<br>285.07 → 239.12 (35)
Apigenin [c]	520-36-5	-	12.31	269.09 → 117.12 (37)<br>269.09 → 149.12 (26)<br>269.09 → 151.06 (26)
Chrysin [e]	480-40-0	+	13.05	253.13 → 143.18 (30)<br>253.13 → 63.20 (34)

	253.13 → 145.16 (31)

[a] Underlined MS/MS transition used for quantification purpose. [b] Sigma Aldrich GmbH (Steinheim, Germany), [c] Extrasynthese (Genay, France), [d] TCI (Tokyo Chemical Industry) (Tokyo, Japan), [e] Alpha Aesar (Kandel, Germany), [f] Anthocyanidins detection after acid hydrolysis.

Table S2. LC-MS/MS performance for the 22 polyphenols identified in the blueberry extracts.

Compound	R^2	Calibration curve	IDL ($\mu g\ L^{-1}$) [a]
Gallic acid	0.9972	y = 3331.7x + 230025	0.93
3,4-dihydroxybenzoic acid	0.9975	y = 3487.2x - 132653	0.14
3,4-dihydroxybenzaldehyde	9.9982	y = 1967.4x + 1E+6	1.07
Procyanidin B1	0.9960	y = 3184.4x + 140717	0.11
Catechin	0.9995	y = 1496x - 31807	27.6
Procyanidin B2	0.9979	y = 625x - 48344	47.4
Chlorogenic acid	0.9956	y = 16186x - 891959	0.75
Caffeic Acid	0.9953	y= 39450x + 2E+7	1.3
Procyanidin C1	0.9902	y = 42.606x + 17205	16.3
Epicatechin	0.9980	y = 1941.7x + 426449	17.8
Petunidin-3-O-glucoside	0.9998	y = 9E+7x – 1E+7	11.4
Epicatechingallate	0.9901	y = 954.26x - 623942	30.3
Procyanidin A2	0.9993	y = 21030x – 3E+6	1.26
Delphynidin	0.9928	y = 4E+6 + 26225	467.15
Cyanidin	0.9897	y = 4E+6 - 23912	415.6
Quercetin-3-rutinoside	0.9987	y = 1120.7x + 7982.5	1.38
Quercetin-3-glucoside	0.9920	y = 236.35x + 73648	2.68
Petunidin	0.9895	y = 7E+6 + 200420	71.1
Myricetin	0.9974	y = 1279.5x + 189066	25.6
Peonidin	0.9998	y = 3E+7 + 494341	33.1
Malvidin	0.9957	y = 8E+6x – 2E+7	293.3
Quercetin	0.9976	y = 2973.1x - 911531	12.01

[a] IDLs were calculated as the concentration giving a signal-to-noise ratio of three (S/N = 3) in all cases, since none of the target compounds were detected in the mobile phase chromatographic blanks
